# Crystal structure of tris­(3-methyl-1*H*-pyrazol-1-yl)methane

**DOI:** 10.1107/S2056989015017247

**Published:** 2015-10-03

**Authors:** Margaret A. Goodman, M. Scott Goodman, Alexander Y. Nazarenko, Ealin N. Patel

**Affiliations:** a Chemistry Department, D’Youville College, 320 Porter Avenue, Buffalo, NY 14201, USA; b Chemistry Department, SUNY Buffalo State, 1300 Elmwood Ave, Buffalo, NY 14222, USA

**Keywords:** crystal structure, 1,1′,1′′-methane­triyltris(3-methyl-1*H*-pyrazole), tripyrazolyl­methane

## Abstract

The title mol­ecule, C_13_H_16_N_6_, crystallizes from hexane as a mol­ecular crystal with no strong inter­molecular inter­actions (the shortest C—H⋯N contact is longer than 3.38 Å). A relatively short intra­molecular contact (3.09 Å) has a C—H⋯N angle of 118° which is quite small to be still considered a hydrogen bond. The three pyrazole rings form a propeller-like motif, with one methylpyrazole unit almost perpendicular to the mean plane of the three rings [82.20 (6)°]. The other two methylpyrazole units, with nitrogen donor atoms oriented in opposite directions, are oriented at 67.26 (6) and 72.53 (6)° to the mean plane.

## Related literature   

For syntheses and reactions of tris­pyrazolyl­methanes and their complexes with transition metals, see: Goodman *et al.* (2012 ▸); Jameson & Castellano (1998 ▸); Reger *et al.* (2000 ▸).
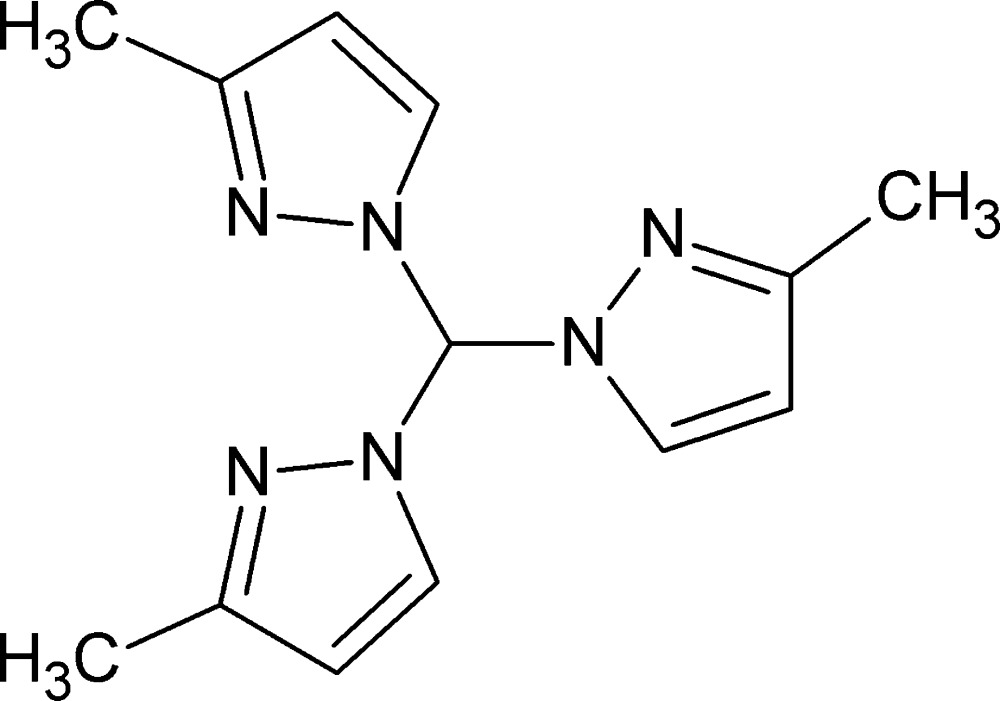


## Experimental   

### Crystal data  

C_13_H_16_N_6_
*M* *_r_* = 256.32
Monoclinic, 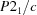

*a* = 12.0881 (8) Å
*b* = 13.4178 (10) Å
*c* = 9.0985 (6) Å
β = 111.630 (2)°
*V* = 1371.82 (16) Å^3^
*Z* = 4
Mo *K*α radiation
μ = 0.08 mm^−1^
*T* = 173 K
0.60 × 0.48 × 0.29 mm

### Data collection  

Bruker Photon-100 CMOS diffractometer
Absorption correction: multi-scan (*SADABS*; Bruker, 2014 ▸) *T*_min_ = 0.706, *T*_max_ = 0.747
22036 measured reflections
2612 independent reflections
2171 reflections with *I* > 2σ(*I*)
*R*_int_ = 0.029

### Refinement  

*R*[*F* ^2^ > 2σ(*F* ^2^)] = 0.037
*wR*(*F* ^2^) = 0.099
*S* = 1.05
2612 reflections
225 parameters
H atoms treated by a mixture of independent and constrained refinement
Δρ_max_ = 0.28 e Å^−3^
Δρ_min_ = −0.19 e Å^−3^

### 

Data collection: *APEX2* (Bruker, 2013 ▸); cell refinement: *SAINT* (Bruker, 2013 ▸); data reduction: *SAINT*; program(s) used to solve structure: *SHELXT* (Sheldrick, 2015*a* ▸); program(s) used to refine structure: *SHELXL2014* (Sheldrick, 2015*b* ▸); molecular graphics: *OLEX2* (Dolomanov *et al.*, 2009 ▸); software used to prepare material for publication: *OLEX2*.

## Supplementary Material

Crystal structure: contains datablock(s) I. DOI: 10.1107/S2056989015017247/zl2643sup1.cif

Structure factors: contains datablock(s) I. DOI: 10.1107/S2056989015017247/zl2643Isup2.hkl

Supporting information file. DOI: 10.1107/S2056989015017247/zl2643Isup3.cdx

Supporting information file. DOI: 10.1107/S2056989015017247/zl2643Isup4.cml

**Fig. 1.**
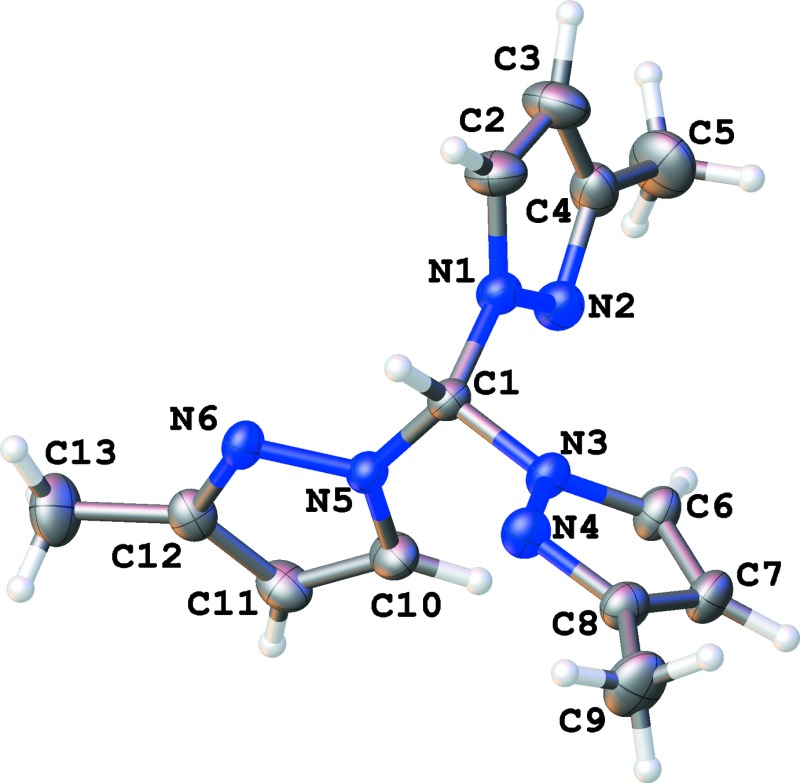
The molecular structure of the title compound. Displacement elipsoids are drawn at the 50% probability level. Disorder of H atoms bonded to C5 are omitted for clarity.

**Fig. 2.**
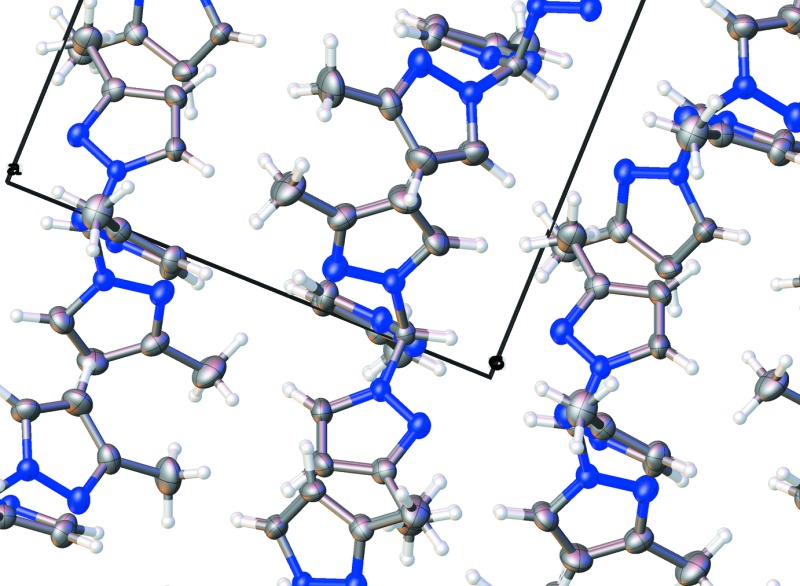
Packing diagram of the title molecule. View along the c axis.

. DOI: 10.1107/S2056989015017247/zl2643fig1.tif
The mol­ecular structure of the title compound. Displacement elipsoids are drawn at the 50% probability level. Disorder of H atoms bonded to C5 are omitted for clarity.

c . DOI: 10.1107/S2056989015017247/zl2643fig2.tif
Packing diagram of the title mol­ecule. View along the *c* axis.

CCDC reference: 1424633

Additional supporting information:  crystallographic information; 3D view; checkCIF report

## supplementary crystallographic information

### S1. Chemical context

This report is part of our continious effort to study substituted tris­pyrazolyl­methanes and their complexes with various metal ions. Because all synthetic procedures yield a complex mixture of isomers, positive identification of the ligand molecule by X-ray diffractometry is essential for future research.

### S2. Refinement

Crystal data, data collection and structure refinement details are summarized in Table 1.

All hydrogen atoms were located in electron difference density Fourier maps and were refined in an isotropic approximation. One methyl group (C5) was treated as disordered (SHELXL instruction AFIX 124). Isotropic parameters of atoms H1 and of disordered methyl group hydrogen atoms were constrained as U_H_ = 1.2 U_C_.

Reflections 1 0 0 and 1 1 0 were too close to the beamstop to be measured reliably and were excluded from refinement.

### S3. Synthesis and crystallization

Following the general method of Reger *et al.* (2000), 3-methyl­pyrazole (6.0 mL, 75.0 mmol), tetra­butyl­ammonium bromide (1.21 g, 3.75 mmol), and sodium carbonate (47.0 g, 0.45 mol) were heated together in a biphasic mixture of deionized water (75 mL) and chloro­form (40 mL). The reaction mixture was allowed to gently reflux for approximately 72 hours under argon. After cooling and filtering, the organic layer was separated from the aqueous layer. The aqueous layer was extracted three times with di­ethyl ether (100 mL), and the combined organic portions were washed twice with 100 mL portions of H_2_O. The organic mixture was dried (Na_2_SO_4_) and the solvents were removed under vacuum to give a dark, brown oil. ^1^H NMR analysis showed this to be mainly a mixture of all four regioisomers of the tris­(pyrazolyl)methanes derived from 3-methyl­pyrazole.

The crude mixture of tris­(pyrazolyl)methane regioisomers was first isomerized according to the method of Jameson & Castellano (1998). The crude brown oil (1.0 g) was combined with a catalytic amount of *p*-toluene­sulfonic acid (0.060 g) and a small amount (50 µL) of 3-methyl­pyrazole and heated at reflux in dry toluene (15 mL) for 24 hours under argon. After cooling, the mixture was washed twice with 100 mL portions of saturated NaHCO_3_ (aq). The aqueous extracts were then extracted once with CH_2_Cl_2_ (100 mL). The organic layers were combined, dried with Na_2_SO_4_, and evaporated under reduced pressure to give a dark yellow oil. NMR analysis of this oil showed that it contained a 2:1 mixture of the desired tris­(pyrazolyl)methane and another regioisomer.

For purification, the material was dissolved in a minimum amount of hot hexane and allowed to crystallize at room temperature for 24 hours. The resulting yellow/brown crystals were separated under a microscope. The larger, clear, and darker-colored lozenges were separated from the smaller, opaque, and lighter plates. These smaller crystals tend to form in masses, often growing on the larger crystals and the bottom of the flask. The larger crystals were scraped clean of as much of the other material as possible under the microscope. The large crystals separated in this fashion were typically at least 85% of target compound. This material was then carefully crystallized from hot hexanes after decolorization with carbon in the same solvent.

A suitable crystal was carefully cut from a larger block. A bigger crystal demonstrated the same structure in a preliminary X-ray experiment.

### Crystal data

**Table:**

C_13_H_16_N_6_	*F*(000) = 544
*M**_r_* = 256.32	*D*_x_ = 1.241 Mg m^−^^3^
Monoclinic, *P*2_1_/*c*	Mo *K*α radiation, λ = 0.71073 Å
*a* = 12.0881 (8) Å	Cell parameters from 9433 reflections
*b* = 13.4178 (10) Å	θ = 2.9–25.7°
*c* = 9.0985 (6) Å	µ = 0.08 mm^−^^1^
β = 111.630 (2)°	*T* = 173 K
*V* = 1371.82 (16) Å^3^	Block, colourless
*Z* = 4	0.60 × 0.48 × 0.29 mm

### Data collection

**Table:**

Bruker Photon-100 CMOS diffractometer	2171 reflections with *I* > 2σ(*I*)
Radiation source: sealedtube	*R*_int_ = 0.029
φ and ω scans	θ_max_ = 25.7°, θ_min_ = 2.9°
Absorption correction: multi-scan (*SADABS*; Bruker, 2014)	*h* = −14→14
*T*_min_ = 0.706, *T*_max_ = 0.747	*k* = −16→16
22036 measured reflections	*l* = −10→11
2612 independent reflections	

### Refinement

**Table:**

Refinement on *F*^2^	0 restraints
Least-squares matrix: full	Hydrogen site location: mixed
*R*[*F*^2^ > 2σ(*F*^2^)] = 0.037	H atoms treated by a mixture of independent and constrained refinement
*wR*(*F*^2^) = 0.099	*w* = 1/[σ^2^(*F*_o_^2^) + (0.0457*P*)^2^ + 0.5131*P*] where *P* = (*F*_o_^2^ + 2*F*_c_^2^)/3
*S* = 1.05	(Δ/σ)_max_ < 0.001
2612 reflections	Δρ_max_ = 0.28 e Å^−^^3^
225 parameters	Δρ_min_ = −0.19 e Å^−^^3^

### Special details

**Table:**

Experimental. SADABS-2014/5 (Bruker,2014/5) was used for absorption correction. wR2(int) was 0.0499 before and 0.0468 after correction. The ratio of minimum to maximum transmission is 0.9453. The λ/2 correction factor is 0.00150.
Geometry. All esds (except the esd in the dihedral angle between two l.s. planes) are estimated using the full covariance matrix. The cell esds are taken into account individually in the estimation of esds in distances, angles and torsion angles; correlations between esds in cell parameters are only used when they are defined by crystal symmetry. An approximate (isotropic) treatment of cell esds is used for estimating esds involving l.s. planes.
Refinement. 1. Fixed Uiso At 1.2 times of: All C(H) groups, All C(H,H,H,H,H,H) groups 2. Others Sof(H5D)=Sof(H5E)=Sof(H5F)=1-FVAR(1) Sof(H5A)=Sof(H5B)=Sof(H5C)=FVAR(1) 3.a Disordered Me refined with riding coordinates and stretchable bonds: C5(H5A,H5B,H5C,H5D,H5E,H5F)

### Fractional atomic coordinates and isotropic or equivalent isotropic displacement parameters (Å^2^)

**Table:**

	*x*	*y*	*z*	*U*_iso_*/*U*_eq_	Occ. (<1)
N1	0.75721 (10)	0.58635 (9)	0.89470 (13)	0.0257 (3)	
C1	0.81554 (12)	0.51681 (10)	0.82664 (15)	0.0231 (3)	
H1	0.8972 (14)	0.5366 (11)	0.8603 (18)	0.028*	
N2	0.64449 (11)	0.57102 (10)	0.88717 (14)	0.0322 (3)	
C2	0.80352 (15)	0.67251 (12)	0.96750 (19)	0.0379 (4)	
H2	0.8860 (18)	0.6925 (14)	0.984 (2)	0.051 (5)*	
N3	0.76487 (10)	0.52045 (9)	0.65612 (13)	0.0247 (3)	
C3	0.71772 (17)	0.71542 (14)	1.0092 (2)	0.0469 (4)	
H3	0.7246 (19)	0.7797 (18)	1.066 (3)	0.074 (7)*	
N4	0.83993 (11)	0.50592 (9)	0.57737 (14)	0.0288 (3)	
C4	0.62038 (14)	0.65090 (12)	0.95767 (18)	0.0358 (4)	
N5	0.81296 (9)	0.41679 (8)	0.88404 (12)	0.0224 (3)	
C5	0.50296 (17)	0.66100 (16)	0.9753 (2)	0.0547 (5)	
H5A	0.50076 (17)	0.7279 (9)	1.0330 (8)	0.066*	0.544 (19)
H5B	0.4350 (9)	0.66087 (16)	0.8637 (14)	0.066*	0.544 (19)
H5C	0.4909 (2)	0.6013 (8)	1.0416 (9)	0.066*	0.544 (19)
H5D	0.4504 (7)	0.5989 (8)	0.9259 (7)	0.066*	0.456 (19)
H5E	0.5161 (2)	0.66585 (18)	1.0952 (15)	0.066*	0.456 (19)
H5F	0.4602 (6)	0.7254 (8)	0.9172 (8)	0.066*	0.456 (19)
N6	0.90717 (10)	0.38708 (9)	1.01297 (12)	0.0249 (3)	
C6	0.64987 (13)	0.52449 (12)	0.55706 (17)	0.0310 (3)	
H6	0.5895 (15)	0.5348 (12)	0.5961 (19)	0.031 (4)*	
C7	0.64953 (14)	0.51349 (12)	0.40812 (17)	0.0332 (4)	
H7	0.5830 (16)	0.5140 (13)	0.313 (2)	0.043 (5)*	
C8	0.76915 (13)	0.50248 (11)	0.42596 (16)	0.0299 (3)	
C9	0.82000 (19)	0.48816 (18)	0.3011 (2)	0.0473 (5)	
H9A	0.905 (2)	0.4833 (17)	0.347 (3)	0.072 (7)*	
H9B	0.784 (2)	0.433 (2)	0.236 (3)	0.084 (8)*	
H9C	0.805 (2)	0.548 (2)	0.234 (3)	0.088 (8)*	
C10	0.72423 (13)	0.34908 (11)	0.84262 (18)	0.0311 (3)	
H10	0.6507 (15)	0.3624 (12)	0.755 (2)	0.036 (4)*	
C11	0.76199 (14)	0.27160 (12)	0.94568 (19)	0.0332 (4)	
H11	0.7205 (15)	0.2122 (13)	0.945 (2)	0.039 (5)*	
C12	0.87610 (13)	0.29819 (11)	1.04977 (16)	0.0285 (3)	
C13	0.95886 (18)	0.24190 (16)	1.1873 (2)	0.0454 (4)	
H13A	1.032 (2)	0.2793 (19)	1.244 (3)	0.086 (8)*	
H13B	0.921 (2)	0.2225 (19)	1.253 (3)	0.088 (8)*	
H13C	0.982 (2)	0.183 (2)	1.150 (3)	0.094 (9)*	

### Atomic displacement parameters (Å^2^)

**Table:**

	*U*^11^	*U*^22^	*U*^33^	*U*^12^	*U*^13^	*U*^23^
N1	0.0252 (6)	0.0268 (6)	0.0242 (6)	−0.0002 (5)	0.0081 (5)	−0.0029 (5)
C1	0.0219 (7)	0.0251 (7)	0.0210 (7)	−0.0020 (5)	0.0063 (5)	−0.0013 (5)
N2	0.0272 (7)	0.0379 (7)	0.0315 (7)	0.0033 (5)	0.0108 (5)	−0.0050 (5)
C2	0.0404 (10)	0.0334 (9)	0.0411 (9)	−0.0069 (7)	0.0163 (7)	−0.0108 (7)
N3	0.0224 (6)	0.0305 (7)	0.0209 (6)	−0.0011 (5)	0.0077 (5)	−0.0003 (5)
C3	0.0528 (11)	0.0350 (10)	0.0560 (11)	0.0016 (8)	0.0237 (9)	−0.0134 (8)
N4	0.0281 (7)	0.0350 (7)	0.0261 (6)	0.0001 (5)	0.0134 (5)	0.0006 (5)
C4	0.0351 (9)	0.0406 (9)	0.0316 (8)	0.0104 (7)	0.0122 (7)	0.0000 (7)
N5	0.0199 (6)	0.0241 (6)	0.0221 (6)	−0.0007 (4)	0.0064 (4)	−0.0015 (5)
C5	0.0450 (11)	0.0647 (13)	0.0602 (12)	0.0151 (9)	0.0260 (9)	−0.0059 (10)
N6	0.0231 (6)	0.0310 (7)	0.0207 (6)	0.0004 (5)	0.0081 (5)	0.0023 (5)
C6	0.0236 (7)	0.0424 (9)	0.0252 (7)	−0.0006 (6)	0.0070 (6)	−0.0003 (6)
C7	0.0317 (8)	0.0409 (9)	0.0224 (7)	−0.0006 (7)	0.0048 (6)	0.0004 (6)
C8	0.0347 (8)	0.0309 (8)	0.0245 (7)	−0.0015 (6)	0.0115 (6)	0.0007 (6)
C9	0.0467 (11)	0.0697 (14)	0.0302 (9)	−0.0003 (10)	0.0195 (8)	−0.0020 (9)
C10	0.0236 (8)	0.0287 (8)	0.0374 (8)	−0.0033 (6)	0.0069 (7)	−0.0032 (6)
C11	0.0314 (8)	0.0262 (8)	0.0455 (9)	−0.0045 (6)	0.0182 (7)	0.0001 (7)
C12	0.0302 (8)	0.0309 (8)	0.0295 (7)	0.0018 (6)	0.0169 (6)	0.0040 (6)
C13	0.0448 (11)	0.0479 (11)	0.0436 (10)	0.0026 (9)	0.0163 (9)	0.0205 (9)

### Geometric parameters (Å, º)

**Table:**

N1—C1	1.4398 (18)	C5—H5D	1.045 (13)
N1—N2	1.3546 (17)	C5—H5E	1.045 (13)
N1—C2	1.347 (2)	C5—H5F	1.045 (13)
C1—H1	0.956 (16)	N6—C12	1.3298 (19)
C1—N3	1.4436 (17)	C6—H6	0.932 (17)
C1—N5	1.4446 (17)	C6—C7	1.362 (2)
N2—C4	1.3353 (19)	C7—H7	0.939 (18)
C2—H2	0.99 (2)	C7—C8	1.402 (2)
C2—C3	1.357 (2)	C8—C9	1.490 (2)
N3—N4	1.3615 (16)	C9—H9A	0.96 (2)
N3—C6	1.3506 (18)	C9—H9B	0.95 (3)
C3—H3	0.99 (2)	C9—H9C	0.98 (3)
C3—C4	1.395 (2)	C10—H10	0.965 (17)
N4—C8	1.3278 (18)	C10—C11	1.361 (2)
C4—C5	1.492 (2)	C11—H11	0.941 (18)
N5—N6	1.3591 (15)	C11—C12	1.401 (2)
N5—C10	1.3490 (18)	C12—C13	1.488 (2)
C5—H5A	1.045 (13)	C13—H13A	0.98 (3)
C5—H5B	1.045 (13)	C13—H13B	0.92 (3)
C5—H5C	1.045 (13)	C13—H13C	0.95 (3)
			
N2—N1—C1	121.52 (11)	H5B—C5—H5E	141.1
C2—N1—C1	125.94 (12)	H5B—C5—H5F	56.3
C2—N1—N2	112.52 (12)	H5C—C5—H5D	56.3
N1—C1—H1	107.0 (9)	H5C—C5—H5E	56.3
N1—C1—N3	111.06 (11)	H5C—C5—H5F	141.1
N1—C1—N5	111.55 (11)	H5D—C5—H5E	109.5
N3—C1—H1	108.3 (9)	H5D—C5—H5F	109.5
N3—C1—N5	111.27 (11)	H5E—C5—H5F	109.5
N5—C1—H1	107.4 (9)	C12—N6—N5	104.82 (11)
C4—N2—N1	104.33 (12)	N3—C6—H6	120.6 (10)
N1—C2—H2	121.4 (11)	N3—C6—C7	106.53 (13)
N1—C2—C3	106.24 (15)	C7—C6—H6	132.8 (10)
C3—C2—H2	132.4 (11)	C6—C7—H7	127.0 (11)
N4—N3—C1	117.37 (11)	C6—C7—C8	105.75 (13)
C6—N3—C1	130.02 (12)	C8—C7—H7	127.2 (11)
C6—N3—N4	112.10 (11)	N4—C8—C7	111.04 (13)
C2—C3—H3	126.0 (13)	N4—C8—C9	120.42 (14)
C2—C3—C4	106.23 (15)	C7—C8—C9	128.53 (14)
C4—C3—H3	127.8 (13)	C8—C9—H9A	110.7 (14)
C8—N4—N3	104.56 (11)	C8—C9—H9B	110.7 (15)
N2—C4—C3	110.68 (14)	C8—C9—H9C	109.5 (15)
N2—C4—C5	120.61 (16)	H9A—C9—H9B	113 (2)
C3—C4—C5	128.69 (16)	H9A—C9—H9C	104.5 (19)
N6—N5—C1	117.49 (11)	H9B—C9—H9C	108 (2)
C10—N5—C1	130.21 (12)	N5—C10—H10	120.1 (10)
C10—N5—N6	111.75 (11)	N5—C10—C11	106.96 (13)
C4—C5—H5A	109.5	C11—C10—H10	132.9 (10)
C4—C5—H5B	109.5	C10—C11—H11	127.0 (10)
C4—C5—H5C	109.5	C10—C11—C12	105.53 (13)
C4—C5—H5D	109.5	C12—C11—H11	127.5 (10)
C4—C5—H5E	109.5	N6—C12—C11	110.93 (13)
C4—C5—H5F	109.5	N6—C12—C13	120.15 (14)
H5A—C5—H5B	109.5	C11—C12—C13	128.92 (15)
H5A—C5—H5C	109.5	C12—C13—H13A	112.2 (15)
H5A—C5—H5D	141.1	C12—C13—H13B	110.5 (16)
H5A—C5—H5E	56.3	C12—C13—H13C	108.8 (16)
H5A—C5—H5F	56.3	H13A—C13—H13B	112 (2)
H5B—C5—H5C	109.5	H13A—C13—H13C	107 (2)
H5B—C5—H5D	56.3	H13B—C13—H13C	106 (2)
			
N1—C1—N3—N4	145.25 (12)	C2—C3—C4—C5	178.91 (17)
N1—C1—N3—C6	−43.68 (19)	N3—C1—N5—N6	142.82 (11)
N1—C1—N5—N6	−92.55 (13)	N3—C1—N5—C10	−46.46 (19)
N1—C1—N5—C10	78.17 (17)	N3—N4—C8—C7	−0.73 (16)
N1—N2—C4—C3	−0.18 (17)	N3—N4—C8—C9	179.43 (15)
N1—N2—C4—C5	−179.06 (15)	N3—C6—C7—C8	0.29 (18)
N1—C2—C3—C4	−0.05 (19)	N4—N3—C6—C7	−0.78 (17)
C1—N1—N2—C4	−178.37 (12)	N5—C1—N3—N4	−89.84 (14)
C1—N1—C2—C3	178.37 (14)	N5—C1—N3—C6	81.22 (18)
C1—N3—N4—C8	173.56 (12)	N5—N6—C12—C11	−0.56 (15)
C1—N3—C6—C7	−172.22 (14)	N5—N6—C12—C13	180.00 (14)
C1—N5—N6—C12	173.41 (11)	N5—C10—C11—C12	0.69 (16)
C1—N5—C10—C11	−172.24 (13)	N6—N5—C10—C11	−1.10 (16)
N2—N1—C1—N3	71.12 (16)	C6—N3—N4—C8	0.94 (16)
N2—N1—C1—N5	−53.63 (16)	C6—C7—C8—N4	0.29 (18)
N2—N1—C2—C3	−0.06 (18)	C6—C7—C8—C9	−179.89 (18)
C2—N1—C1—N3	−107.19 (16)	C10—N5—N6—C12	1.03 (15)
C2—N1—C1—N5	128.06 (15)	C10—C11—C12—N6	−0.07 (17)
C2—N1—N2—C4	0.15 (16)	C10—C11—C12—C13	179.30 (17)
C2—C3—C4—N2	0.1 (2)		

### Hydrogen-bond geometry (Å, º)

**Table:**

*D*—H···*A*	*D*—H	H···*A*	*D*···*A*	*D*—H···*A*
C1—H1···N6^i^	0.96 (2)	2.44 (2)	3.3796 (19)	167 (1)
C6—H6···N2	0.932 (17)	2.529 (16)	3.0918 (19)	119.2 (13)

Symmetry code: (i) −*x*+2, −*y*+1, −*z*+2.
