# Supplementary figures and images for: Crystal structure of tris­(3-methyl-1H-pyrazol-1-yl)methane
Source: Acta Crystallogr E Crystallogr Commun. 2015 Oct 3;71(Pt 11):o816. doi: 10.1107/S2056989015017247 (PMC4645007; doi:10.1107/S2056989015017247)

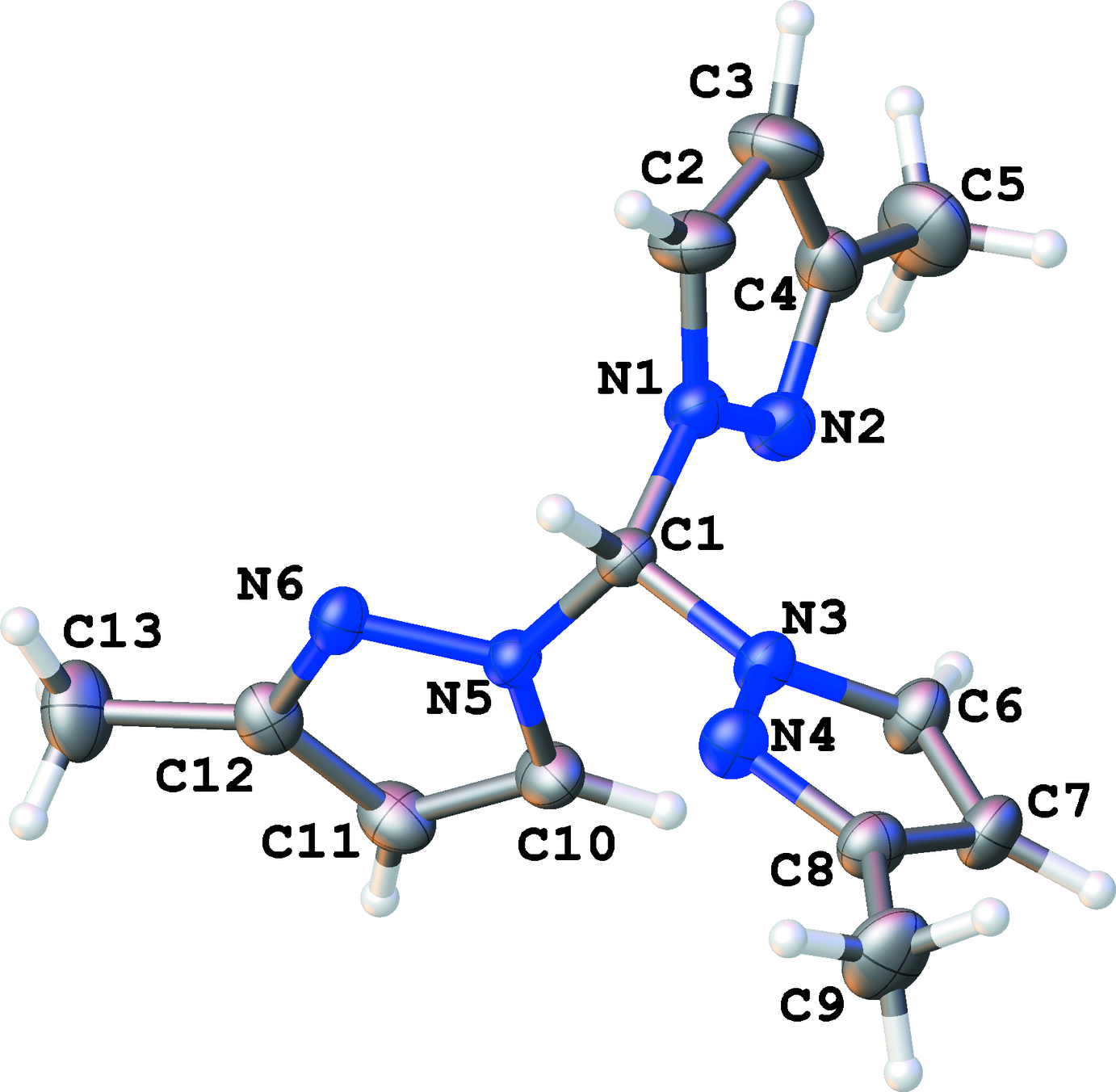

Supplement: Supplementary file 5 [file e-71-0o816-fig1.tif]

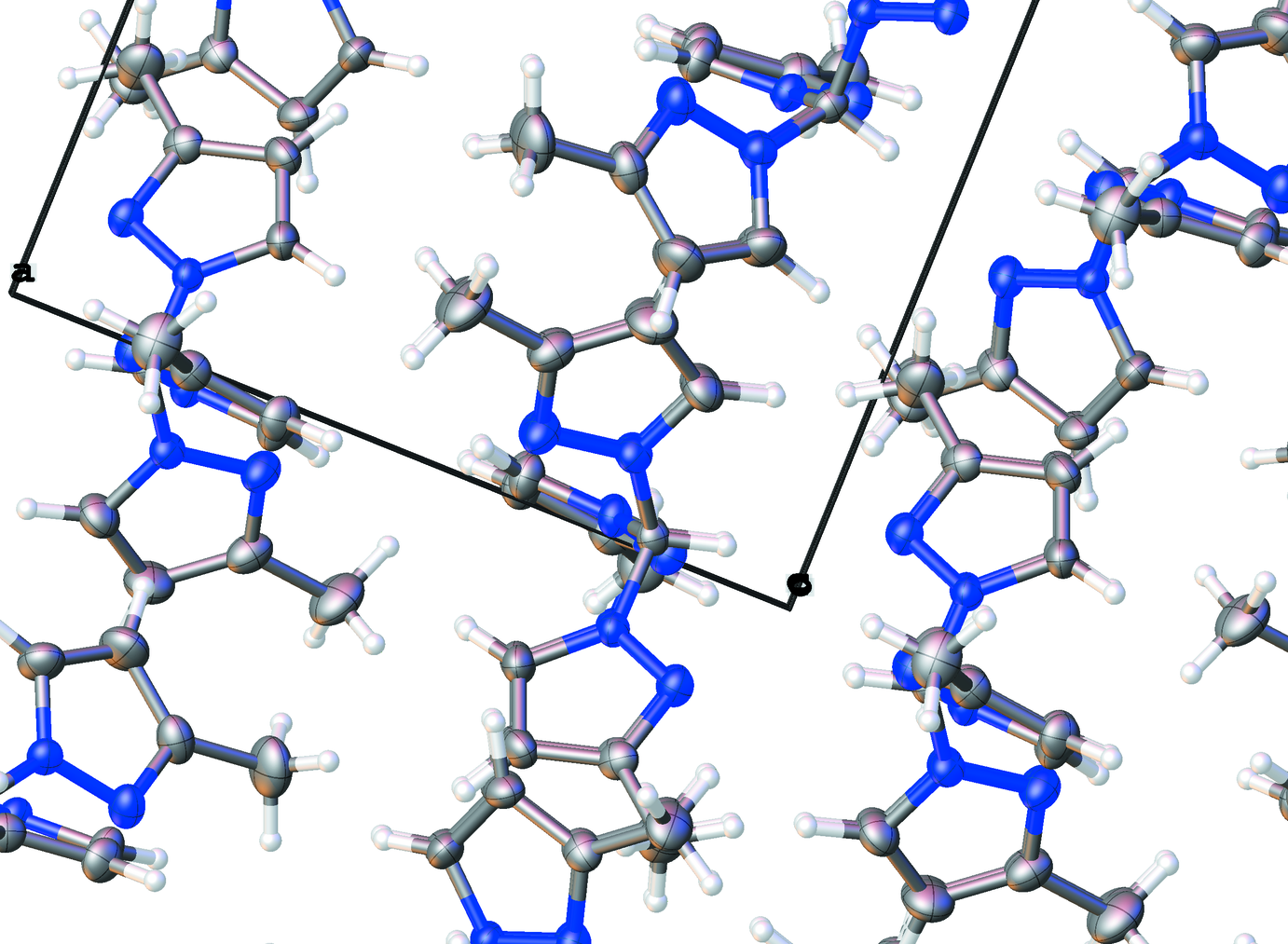

Supplement: Supplementary file 6 [file e-71-0o816-fig2.tif]
